# Evaluating Crossbred Red Rice Variants for Postprandial Glucometabolic Responses: A Comparison with Commercial Varieties

**DOI:** 10.3390/nu8050308

**Published:** 2016-05-20

**Authors:** Chee-Hee Se, Khun-Aik Chuah, Ankitta Mishra, Ratnam Wickneswari, Tilakavati Karupaiah

**Affiliations:** 1 School of Healthcare Sciences, Faculty of Health Sciences, National University of Malaysia (UKM), Jalan Raja Muda Abdul Aziz, Kuala Lumpur 50300, Malaysia; jeremysch3232@gmail.com (C.-H.S.); cruise_chuah@hotmail.com (K.-A.C.); 2 School of Environmental and Natural Resource Sciences, Faculty of Science and Technology, National University of Malaysia (UKM), Bangi, Selangor 43600, Malaysia; ankitta99@gmail.com (A.M.); wicki@ukm.edu.my (R.W.)

**Keywords:** red rice, glycaemic index, insulin resistance, peptide hormones, cross-breeding

## Abstract

Consumption of white rice predisposes some Asian populations to increased risk of type 2 diabetes. We compared the postprandial glucometabolic responses to three newly-developed crossbred red rice variants (UKMRC9, UKMRC10, UKMRC11) against three selected commercial rice types (Thai red, Basmati white, Jasmine white) using 50-g carbohydrate equivalents provided to 12 normoglycaemic adults in a crossover design. Venous blood was drawn fasted and postprandially for three hours. Glycaemic (GI) and insulin (II) indices, incremental areas-under-the-curves for glucose and insulin (IAUC_ins_), indices of insulin sensitivity and secretion, lactate and peptide hormones (motilin, neuropeptide-Y, orexin-A) were analyzed. The lowest to highest trends for GI and II were similar *i.e.*, UKMRC9 < Basmati < Thai red < UKMRC10 < UKMRC11 < Jasmine. Postprandial insulinaemia and IAUC_ins_ of only UKMRC9 were significantly the lowest compared to Jasmine. Crude protein and fiber content correlated negatively with the GI values of the test rice. Although peptide hormones were not associated with GI and II characteristics of test rice, early and late phases of prandial neuropeptide-Y changes were negatively correlated with postprandial insulinaemia. This study indicated that only UKMRC9 among the new rice crossbreeds could serve as an alternative cereal option to improve diet quality of Asians with its lowest glycaemic and insulinaemic burden.

## 1. Introduction

Conventional rice breeding programs have long focussed on developing new rice varieties with improved yield-associated traits and micronutrient capacity to meet food security and nutritional needs of developing countries in Asia [1]. However, epidemiologic evidence suggests that greater white rice intake was associated with significantly higher odds of developing metabolic syndrome and type 2 diabetes in Asian Chinese and Japanese populations [2]. The high glycaemic index (GI) of rice renders this food staple the major contributor of glycaemic load in Asian diets. However, disparities in GI values exist even for the same variety of rice [3]. The glycaemic variability can be largely attributed to the inherent starch characteristics of specific cultivars, although within a given rice variety, the mode of post-harvesting processing and at-home preparation also bear considerable influence on starch digestibility [4,5].

Rice improvement programs have not considered the development of varieties targeting the prevention and management of type 2 diabetes because the genetics of GI remain unclear. Natural polymorphisms in the starch biosynthesis related genes, such as *granule bound starch synthase I* (*Waxy*), *branching enzyme I* and *glucose 6-phosphate translocator* could potentially modulate the glycaemic potencies of rice by changing its amylose content and retrogradation rate [6]. A large-scale phenotyping of 235 rice varieties found that *Waxy* gene was the strongest predictor for GI variability [7]. Therefore, lowering the GI characteristic of this dietary staple via breeding technologies could improve the glycaemic burden and diet quality of rice-based diets in Asians [4]. To date, new white rice varieties with lower GI values have been produced through marker-assisted breeding [8] or by increasing resistant starch content via genetic modification [9,10].

In Malaysia, three new transgressive variants with red pericarp grain were derived from advanced backcrosses between a wild rice accession, *Oryza rufipogon* Griff. IRGC105491 and a Malaysian high-yielding rice cultivar, *Oryza sativa* L. subsp. *indica* cv. MR219 [11]. According to a Distinctiveness, Uniformity and Stability test for evaluating *New Plant Variety* status (Protection of New Plant Varieties Act 2004), UKMRC9 and UKMRC10 were different but UKMRC9 and UKMRC11 were similar for 58 morphological and physiological traits [12]. Experimental field trials and physicochemical analyses have confirmed the superiority of these red rice variants in yield potential, resistance against blast disease and antioxidant properties compared to MR219 [13,14]. Karupaiah *et al.* [15] previously experimented with UKMRC9 and found it had low glycaemic and insulinogenic properties, but these favorable traits were lost upon polishing.

In this study, postprandial glucometabolic evaluations were applied to a wider range of red rice variants (UKMRC10, UKMRC11) and compared against imported specialty rice varieties, namely Basmati white, Jasmine white and Thailand red rice. As secondary outcomes, we have evaluated the postprandial effect of these rice types on plasma lactate and peptide hormones. The inclusion of lactate was in light of recent observations relating elevated fasting plasma lactate with various metabolic aberrations, such as insulin resistance, type 2 diabetes and reduced oxidative capacity [16]. Therefore, the key questions addressed in this study were:

- Do the related crossbred red rice variants reflect similar glycaemic and insulin indices (II) with UKMRC9?
- What is the relationship between nutrient content and cooking characteristics of the six rice types with the GI and II characteristics?
- Does consumption of rice with varying GI values have a role to play in modulating postprandial insulin sensitivity, pancreatic β-cell function and peptide hormones?

## 2. Materials and Methods

### 2.1. Test and Reference Food

The wild parent *Oryza rufipogon* Griff. (IRGC105491) and *Oryza sativa* L. subsp. *indica* cv. MR219, a Malaysian high-yielding rice cultivar were backcrossed to produce advanced breeding lines (BC_2_F_5_ and BC_2_F_6_ generations) where transgressive variants with red pericarp grain and higher grain yield were detected [11]. The three red pericarp genotypes tested in this study are registered as *New Plant Variety* under the Ministry of Agriculture, Malaysia, namely UKMRC9 (PBR 0032), UKMRC10 (PBR 0033) and UKMRC11 (PVBT041/09). Additionally, Basmati white rice (IndiaGate, KRBL Ltd., Hyderabad, India), Thailand red rice (NutriRice, Jasmine Food Corp. Pte. Ltd., Kuala Lumpur, Malaysia) and Jasmine white rice (Rambutan AAA, Jasmine Food Corp. Pte. Ltd., Kuala Lumpur, Malaysia) were the commercial varieties tested. All red rice varieties were tested in dehusked form whilst Basmati and Jasmine rice were polished. For each rice type, different packaged samples were mixed together as a single sample for homogeneity, before re-packaging into 500 g portions and then storing at 2 ± 1 °C.

Fifty grams of unflavoured dextrose monohydrate were dissolved in 250 mL water and served as the reference food (Glucolin™, The Boots Company, Nottingham, UK). Glucose standard was tested twice at the first and last feeding sessions to obtain an average value for the determination of plasma glucose and insulin standards [17].

### 2.2. Chemical Composition of Rice

Rice samples were analyzed for crude protein (method 981.10), fat (method 991.36), moisture (method 950.46), ash (method 923.03) and total dietary fiber (method 991.43) content by the Association of Official Analytical Chemists methods [18]. Total carbohydrate content was calculated by proximate difference [19]. Total energy content was calculated by multiplying the nutrient content with Atwater conversion factors 4, 9 and 4 kcal/g for protein, fat and carbohydrate respectively. Amylose content was determined using the iodine colorimetric method [20]. Total phenolic content was evaluated using the Folin–Ciocalteu method [14].

### 2.3. Rice Preparation for Postprandial Testing

The rice was cooked to completion in an electronic rice cooker (Panasonic model SR-WN36, Osaka, Japan) using a consistent rice-to-water ratio of 1:2 (*w*/*w*). Rice samples were portioned and weighed when cooled to room temperature according to 50 g available carbohydrate content [19].

### 2.4. Subject Recruitment and Screening Procedures

Twelve non-obese subjects (5 men and 7 women), aged 21–40 years and without history of chronic disease(s) were enrolled into the study. Exclusion criteria included pregnant or nursing women or subjects receiving pharmacotherapy that would interfere with glucose metabolism, smokers, consuming alcohol or on low-calorie diets. Eligible participants underwent baseline medical, dietary practices and blood chemistry screening. Their baseline characteristics were as follows (mean ± SD): age = 23.2 ± 1.4 years; body mass index = 22.1 ± 3.1 kg/m^2^; fasting plasma glucose = 5.02 ± 0.32 mmol/L; fasting plasma insulin = 6.17 ± 2.07 mU/L. None of the subjects were insulin resistant as their individual homeostatic model assessment of insulin resistance (HOMA-IR) scores were below 2.6.

### 2.5. Experimental Protocol

A crossover design was adopted with all subjects completing eight postprandial evaluations on separate mornings with a one-week washout period between rotations. Before each test rotation, subjects refrained from strenuous physical activity or sport games and maintained their customary dietary intake for 48 h prior to testing days. On testing days, fasted subjects reported to the feeding laboratory between 07:00 and 08:00 h. Subjects rested for 15 min prior to blood collection. Fasting blood samples (0 min) were drawn before subjects consumed 50 g carbohydrate equivalents of test rice with 250 mL of plain water within 10 min. Subjects continued resting until all postprandial blood samplings at 15, 30, 45, 60, 90, 120 and 180 min were completed. The study protocol was approved by the Research Ethics Committee of Universiti Kebangsaan Malaysia (registration No.: NN-069-2012) and written informed consent was obtained from all subjects prior to study commencement.

### 2.6. Blood Sampling, Processing and Storage Procedures

Fasting and postprandial venous blood samples were obtained via antecubital venipuncture, with the subject’s arm alternated for each sequential blood draw. Blood samples were collected into three evacuated Vacutainer^®^ tubes containing ethylenediaminetetraacetic acid (0.117 mL of 15% EDTA), lithium heparin or sodium fluoride-potassium oxalate, respectively (Becton Dickinson Vacutainer, Franklin Lakes, NJ, USA). The tubes were centrifuged for 10 min at 3000 rpm and collected plasma was subsequently aliquoted, snap-frozen using liquid nitrogen and then stored at −80 °C for later analysis.

### 2.7. Biochemical Analyses

[i]. Plasma glucose: Plasma glucose concentrations (mmol/L) were quantified using a Roche Modular P800 (Roche Diagnostics, Tokyo, Japan) automated analyzer by the enzymatic hexokinase method [21]. The assay had a detection limit of 0.11 mmol/L and the intra- and inter-assay coefficients of variation (CV) were <2.0%.
[ii]. Plasma insulin: Heparinized plasma samples were analyzed for insulin concentrations (mU/L) using electrochemiluminescence immunoassay on the Modular Analytics E170 system (Roche Diagnostics, Tokyo, Japan). The fully-automated assay adopts a solid-phase, two-site, enzyme-labeled immunoassay based on the direct sandwich technique [22]. The intra- and inter-assay CVs were <5%, with a lower detection limit of 0.20 mU/L.
[iii]. Plasma lactate: The plasma L-lactate concentration (mmol/L) was assayed on a Roche Modular P800 analyser (Roche Diagnostics, Tokyo, Japan) using the lactate oxidase method [23]. The assay had a detection range between 0.22 and 15.5 mmol/L and inter-assay CV of 2.0%.
[iv]. Peptide hormones: Plasma concentrations of motilin (EK-045-04), neuropeptide-Y (EK-049-03) and orexin-A (EK-003-30) were determined in duplicate using commercially-available enzyme immunoassay (EIA) kits from Phoenix Pharmaceuticals (Burlingame, CA, USA), as described previously [24]. The enzyme-linked immunosorbent assay (ELISA) was performed according to the manufacturer’s protocol and absorbance was read with a Tecan Infinite M200 microplate reader (Tecan Group Ltd., Mannedorf, Switzerland). Plasma concentrations were calculated using four-parameter non-linear logistic curve fitting (Magellan Data Analysis Software v. 311 for PC, Tecan Group Ltd., Mannedorf, Switzerland). The standard curve plots were generated using the five standard concentrations ranged from 0.01 to 100 ng/mL. The coefficients of determination for standard curves were >0.97.

For the motilin EIA, the minimum detectable concentration was 0.14 ng/mL, the intra- and inter-assay CVs were <5% and <9%, respectively. The neuropeptide-Y EIA had a lower detection limit of 0.09 ng/mL, the intra- and inter-assay CVs of <4% and <8.5%, respectively. The orexin-A EIA had a minimum detectable concentration of 0.22 ng/mL with the intra- and inter-assay CVs of <6% and <10%, respectively.

### 2.8. Outcome Measures

[i]. Quality control: The mean intra-individual CV for glycaemic response after two 50 g glucose standard loads was 21.3%, which was in concordance with the recommended CV < 30% required for precision and accuracy [25].
[ii]. Glucometabolic markers: Kinetic markers of incremental glucose and insulin peaks are defined as maximum increases in plasma glucose and insulin concentrations obtained at any point after a test rice or glucose challenge. Incremental areas-under-the-curves (IAUC), excluding areas beneath fasting values, for plasma glucose, insulin and lactate were calculated geometrically using the trapezoidal method [19]. The GI and II were calculated by dividing the net IAUC generated from the 3 h postprandial plasma glucose-/insulin-timed responses of the test food with that by the standard glucose load (GI and II = 100), with each subject being their own reference [19]. Individual GI or II scores differing from the mean value by >2 standard deviations (outliers) were excluded from the dataset [25].
[iii]. Insulin kinetics: Additionally, indices of postprandial insulin sensitivity (Matsuda index) and pancreatic β-cell function (insulinogenic index [IGI], IGI/HOMA-IR, IGI/fasting plasma insulin [FPI]) were estimated using the following mathematical models [26,27,28]:

(1) HOMA-IR = Fasting insulin (mU/L) × Fasting glucose (mmol/L)/22.5,

(2) Insulinogenic index (IGI) = ∆Insulin_30-0__min_ (pmol/L)/∆Glucose_30-0__min_ (mmol/L),

(3) Matsuda Index = 10000/(G_0_ × I_0_ × G_mean_ × I_mean_)^0.5^,

where G_0_ = baseline glucose concentration (mg/dL); I_0_ = baseline insulin concentration (mU/L); G_mean_ = mean glucose throughout 2 h postprandial (mg/dL); I_mean_ = mean insulin throughout 2 h postprandial (mU/L)

IGI were further adjusted by HOMA-IR (IGI/HOMA-IR) and fasting plasma insulin (IGI/FPI) to account for between-subjects variations in insulin sensitivity.

### 2.9. Statistical Analyses

The crossover design allowed each subject to serve as his/her own control for the eight postprandial rotations. All data were assessed for normality using the Shapiro–Wilk test. Data are presented as mean ± standard error of the mean (SEM) unless otherwise stated.

Postprandial changes in plasma glucose, insulin, lactate and peptide hormones for the six test rice were analyzed using the general linear model (GLM) for repeated measures to examine the time, diet and time *x* diet interaction effects. As the data distribution of peptide hormones across most timed intervals did not meet normality assumption, data were log-transformed prior to GLM analysis. Greenhouse–Geisser correction for degrees of freedom was used when Mauchly’s test of sphericity was significant. Bonferroni-corrected *post hoc* comparisons were used when the main effects were significant. Calculated GI, II, IAUC and insulin kinetics data for each rice type were compared using univariate analysis of variance, followed by Tukey’s *post hoc* test. Magnitude of the significant paired difference was assessed using partial eta-squared (η_p_^2^). Bivariate associations were examined using Pearson’s correlation test. Statistical significance was pre-set at *p* < 0.05. All analyses were computed using SPSS^®^ for Windows™ applications (Version 16.0; SPSS Inc., Chicago, IL, USA).

## 3. Results

### 3.1. Proximate Composition and Cooking Characteristics of Rice

Proximate nutrient composition of the six test rice varieties is presented in Table 1. Crude protein content in UKMRC9 and Basmati were similar to Thai red rice (*p* > 0.05) but significantly higher compared to UKMRC11 and Jasmine (*p* < 0.05). Both Basmati and Jasmine had the lowest crude lipid, total dietary fiber, ash, and phenolic content (all *p* < 0.001). Polished Basmati and Jasmine required relatively shorter duration for cooking to completion, compared to dehusked red rice cultivars, which took more than 40 min.

### 3.2. Glucometabolic Responses

**Figure 1:**
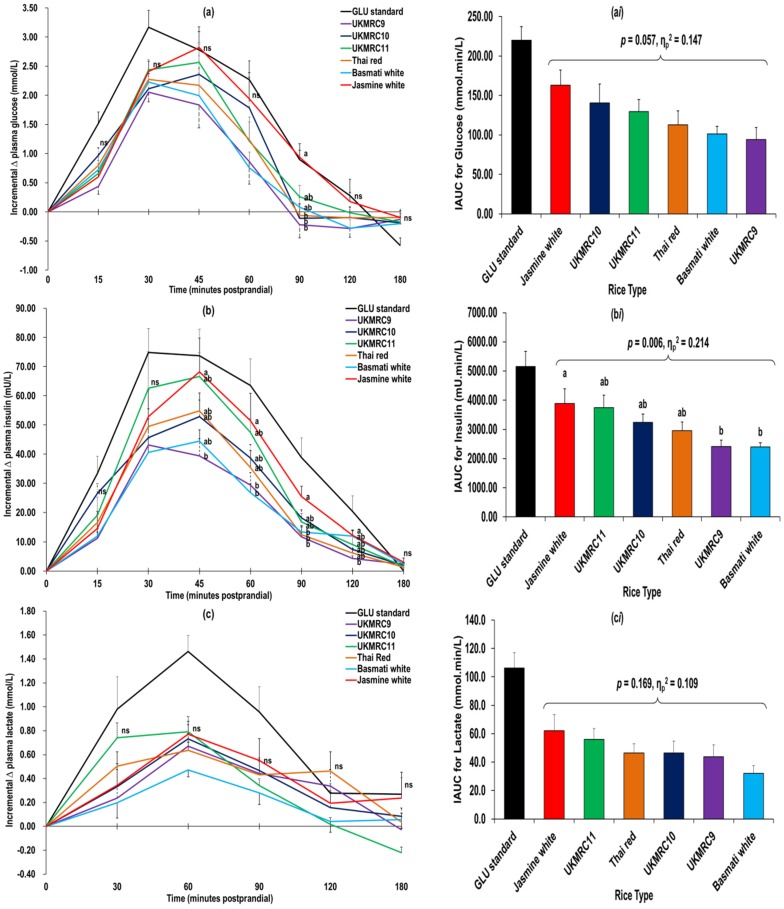
Baseline-adjusted trends (mean ± SEM) in postprandial plasma (**a**) glucose; (**b**) insulin and (**c**) lactate responses for six test rice and glucose standard, and incremental area-under-the-curve (IAUC) for postprandial (**a*i***) glycaemia; (**b*i***) insulinaemia and (**c*i***) lactataemia. ^§^ Mean values bearing the same alphabets were not significantly different (*p* > 0.05, univariate analysis of variance followed by Tukey’s *post hoc* test, ns = not significant); η_p_^2^ = partial eta-squared, 0.01, 0.06 and 0.14 were used to denote small, moderate and large differences in measured outcomes, respectively.

Postprandial glycaemia for the six rice types were not significantly different (*p* = 0.065, η_p_^2^ = 0.143). However, a marginal significance for glycaemic response was noted between UKMRC9 and Jasmine (*p* = 0.056) (Figure 1a), which was also reflected by their IAUC_glu_ (*p* = 0.06) (Figure 1a*i*). UKMRC9 (GI = 46 ± 7.7) and Basmati (GI = 50 ± 5.8) were categorized as low GI, whilst Thai red (GI = 55 ± 8.6), UKMRC10 (GI = 59 ± 8.8) and UKMRC11 (GI = 63 ± 8.6) were categorized as intermediate GI. Jasmine (GI = 77 ± 7.3) was the only test rice in the high GI category (Table 2). GI (*p* = 0.093), maximum (*p* = 0.074) and incremental (*p* = 0.063) glucose peak values were not significantly different between the six rice types. Notably, after consuming Jasmine compared to Basmati, the time taken was significantly greater to reach maximum concentration of plasma glucose (*p* = 0.021).

Postprandial insulinaemic trends of the six test rice were in tandem with their postprandial glycaemic effects (Figure 1b), and diet effects on postprandial insulinaemia were significant (*p* = 0.013, η_p_^2^ = 0.194). *Post hoc* comparison indicated UKMRC9 elicited a marginally significant lower insulin generation compared to UKMRC11 (*p* = 0.083) and Jasmine (*p* = 0.052) (Figure 1b). For IAUC_ins_, Jasmine induced the highest postprandial insulinaemia amongst all rice types, which was significant compared to both Basmati (*p* = 0.032) and UKMRC9 (*p* = 0.033) (Figure 1b*i*). However, the IAUC_ins_ between the crossbred red rice variants were not significantly different, except for a marginal difference between UKMRC11 and UKMRC9 (*p* = 0.069).

Both UKMRC9 (II = 51 ± 5.3, *p* = 0.043) and Basmati (II = 52 ± 5.3, *p* = 0.059) had the lowest II values compared to Jasmine (II = 76 ± 7.1) (Table 2). However, the II values of the three crossbred red rice varieties were not significantly different (*p* > 0.05). A marginal significant difference was observed in the Matsuda index of insulin sensitivity between rice types (*p* = 0.058). Conversely, postprandial pancreatic β-cell function, as modelled by IGI/HOMA-IR and IGI/FPI, did not differ significantly between rice types (*p* > 0.05).

Postprandial lactate responses and IAUC_lac_ were not significantly different between six rice types (*p* > 0.05) (Figure 1c,c*i*).

### 3.3. Correlation between Nutrient Composition, Cooking Characteristics, GI and II

Only crude protein (*r* = −0.357, *p* = 0.002) and total dietary fiber (*r* = −0.237, *p* = 0.047) content correlated negatively with GI values of the six test rice (Table 3), whereas only crude protein was inversely related with II values (*r* = −0.385, *p* = 0.001). No significant correlations were observed between cooking characteristics of test rice with the GI and II properties (*p* > 0.05).

### 3.4. Postprandial Changes in Plasma Motilin, Neuropeptide-Y and Orexin-A

**Figure 2:**
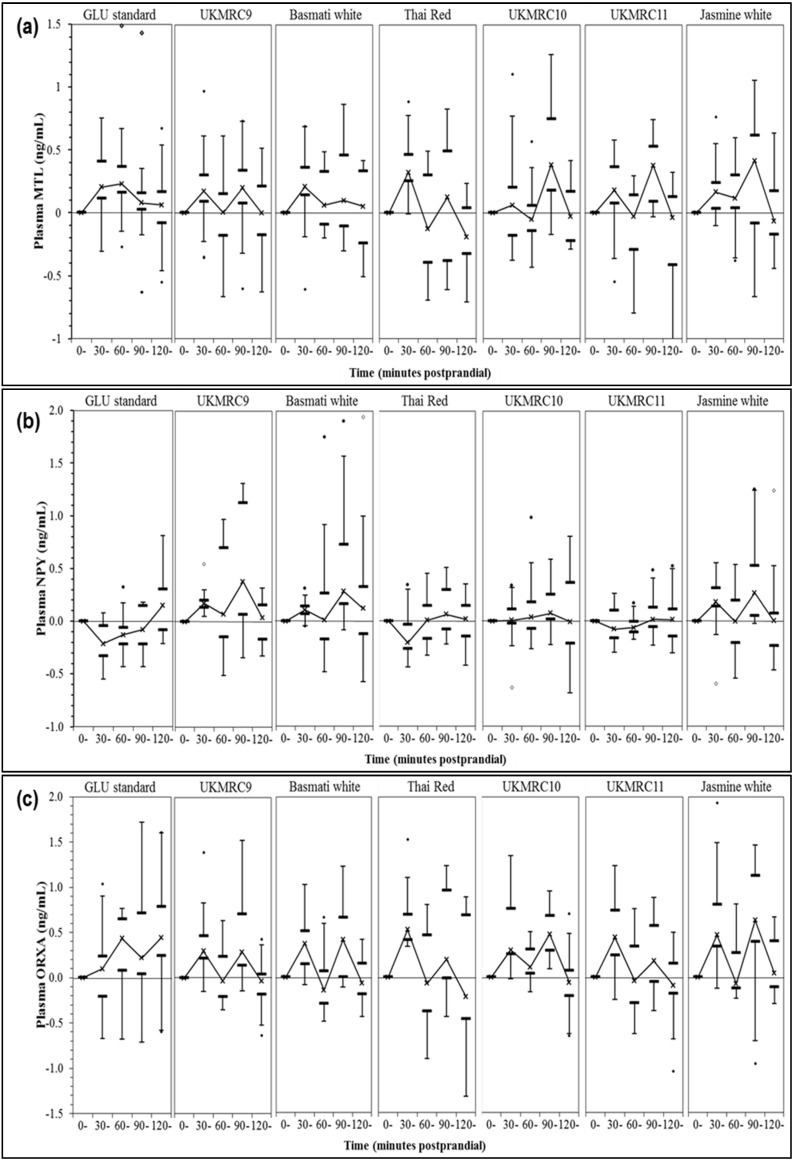
Baseline-adjusted trends in postprandial plasma (**a**) motilin (MTL); (**b**) neuropeptide-Y (NPY) and (**c**) orexin-A (ORXA) responses for six test rice and glucose standard. Note: Horizontal bars = smallest and largest values; Lower band = 25th percentile; Upper band = 75th percentile; (×) = median; (•) = outlier and (◊) = extreme values defined as 1.5 × (Q3 − Q1) below 25th or above 75th percentiles.

Log-transformed values corrected to baseline were reported for plasma motilin, neuropeptide-Y and orexin-A (Figure 2). Biphasic secretory responses peaking at 30- and 90-min were observed for motilin and orexin-A, but between-diet effects were not significant for the six test rice (*p* = 0.804 for motilin; *p* = 0.162 for orexin-A). Contrarily, biphasic neuropeptide-Y responses were only evident after consuming UKMRC9, Basmati and Jasmine. Pairwise comparisons demonstrated that postprandial neuropeptide-Y trend for Thai red differed significantly from UKMRC9, Basmati and Jasmine (all *p* < 0.01) (Supplementary Table S1).

## 4. Discussion

### 4.1. Moderators of GI

This study confirmed the low GI characteristic of UKMRC9, as the mean GI values derived from current and previous studies [15] were similar (46 *vs.* 51). However, despite originating from the same gene pool [13] and having similar amylose content, we found that both UKMRC10 and UKMRC11 did not exhibit low GI properties compared to UKMRC9. Wang [29] was the first to attribute the *Waxy* gene as the regulator of amylose synthesis in rice and Fitzgerald *et al.* [7] proposed that this gene was associated with GI variations in 235 rice samples. However, in view that these crossbred red rice variants had similar amylose content, this study does not corroborate the *Waxy* gene-amylose content link to GI potential of rice.

The glucose-raising potential of high-amylose rice varieties is reported to be lower than rice with greater amylopectin content [7,10,30]. The compact linear chain of amylose has been hypothesized to delay digestion by amylase enzymes in the human gastrointestinal tract, permitting a sustained release of glucose into bloodstream [31]. We did not find any significant relationship between amylose content and GI of test rice, in concordance with some studies [15,31,32], but not all [7,10,30]. The lack of association observed in this study could be attributed to the relatively narrow range of amylose content studied (18%–23%), contrasting with other research comparing the GI values of rice cultivars across a wide range of amylose categories (0%–2% *vs.* >20%) [7,10,30]. However, this study found that the amylose content of Jasmine was higher (23%) compared to those commonly reported, ranging from 12% to 17% [33]. Cooked rice starches with higher amylose content undergo retrogradation upon storage, causing an increase in resistant starch content and lowering of rapidly digestible starch content [34,35]. This could perhaps explain the lower GI value of Jasmine (=77) observed in this study compared to those typically reported as >90 [3]. However, Chiu and Stewart [36] discovered that postprandial glycaemia did not differ significantly in healthy adults after consuming two rice samples with distinctly different resistant starch content (0.20 *vs.* 2.55 g/100 g). This concurs with the observation that GI variability exists within each category of amylose content [7]. It appears that amylose content alone may not be a good predictor of starch digestibility rate and post-meal glycaemia. The current research direction has evolved to investigating the modulatory roles of fine molecular structures of amylose and amylopectin in rice starch digestibility [37].

Additionally, the lower postprandial glycaemic and insulin responses after rice consumption observed in this study were partly mediated by the higher protein and fiber content in rice. Both crude protein and fiber content accounted for about 13% and 6%, respectively, of the GI variations of the six test rice. Physical entrapment of the rice starch granules by the spherical protein bodies located in the sub-aleurone layer may hinder and delay hydrolysis of endosperm starch [38]. Furthermore, *in vitro* studies have demonstrated that the intact bran layer serves as a physical barrier that delays the access of amylase enzymes and gastric acid during starch hydrolysis [39,40]. The GI-increasing effect has been previously reported when both dehusked and polished versions of the same rice type were fed to the same group of subjects [15,41].

### 4.2. Glucometabolic Responses

This study found that the time taken to reach postprandial peak glucose values for Jasmine (high GI) averaged 47.5 min compared to UKMRC9 and Basmati (low GI), which occurred at 37.5 min and 35.0 min, respectively. Moreover, the time needed for glycaemia to return to baseline levels was longer for Jasmine (136.5 min), compared to Basmati (99.6 min) and UKMRC9 (85.8 min), implying that the glucose clearance for high-GI rice required longer duration than those with a low-GI characteristic. These observations paralleled the findings by Brand-Miller *et al.* [42], whereby postprandial glycaemic curves do not differ between low and high GI foods, except for the magnitude of glucose excursions. Indeed, GI values of the six test rice in this study explained 37% and 49% of the variations in postprandial absolute and incremental glucose peaks, respectively. This has important clinical implications, as consumption of high GI foods would therefore translate into greater magnitude of post-meal glycaemic spikes and prolonged glycaemia. This metabolic milieu has been implicated in the pathophysiology of atherogenesis, systemic inflammation and type 2 diabetes [43].

GI (*r* = −0.267, *p* = 0.024) and II (*r* = −0.391, *p* = 0.001) of all test rice correlated negatively with the Matsuda index of insulin sensitivity. Furthermore, although not statistically significant, the large effect size observed in the differences in insulin sensitivity after consuming rice with varying glycaemic impact suggests that rice GI evaluations should factor in postprandial insulin responses. Consumption of lower GI foods or diet at the expense of hyperinsulinaemia may not be metabolically favorable as it has been cited as one of the pathophysiological contributors to the development of chronic diseases, including diabetes [44]. Chronic fluctuations in postprandial glycaemia and insulinaemia were proposed to increase circulating non-esterified fatty acids and reduce the number of insulin receptors, ultimately contributing to insulin resistance [45]. Notably, we found that ingestion of rice with differing GI values did not significantly affect postprandial pancreatic β-cell function. This implies that consumption of a single carbohydrate-rich food *per se* did not significantly alter postprandial insulin secretory capacity.

The glucose standard stimulated the highest early-phase insulin secretion (0–30 min) and was closely followed by UKMRC11, but was not evident in either UKMRC9 or UKMRC10. The early-phase lactate secretion also followed this trend. Surge in plasma glucose concentrations and greater insulin demand after 15 min postprandially is possibly attributed to the release of rapidly digestible starch fractions. Alternately, acute plasma lactate elevations (0–60 min) may contribute to delayed glucose and insulin clearance by peripheral tissues. We found that IAUC_ins_ for the rice evaluations was weakly and positively correlated with IAUC_lac_ as well as incremental and maximum concentrations of plasma lactate (data not shown). Indeed, *in vitro* and *in vivo* rodent studies have elucidated the suppressive effects of increased plasma lactate on glycolytic enzymes, which led to decreased insulin-stimulated glycolysis and sustained postprandial insulinaemia [46,47].

Generation of peptide hormones in response to human physiological digestion of rice alone has never been explored. Peptide hormones, namely motilin, neuropeptide-Y and orexin-A have been investigated in response to GI effects of mixed meals [24]. Wu *et al.* [24] reported that a low GI breakfast reduced the secretion of orexin-A but significantly stimulated motilin secretion, without marked effects on neuropeptide-Y secretion. However, we could not discern any significant differences in all three peptide hormones responses attributed to GI variations of test rice. Fluctuations in plasma motilin and orexin-A observed in this study would perhaps be explained by the form in which carbohydrates were administered, *i.e.*, solid (rice) *versus* liquid (glucose standard). This suggests that consumption of rice with varying GI values may not significantly alter the gastrointestinal motility as well as satiety response.

Both early and late phases of post-meal neuropeptide-Y changes were negatively correlated with postprandial insulinaemia. Insulin enters the brain through the blood-brain barrier and serves to regulate feeding behaviour and metabolism in humans [48]. The early-phase insulin spikes (0–30 min) elicited by UKMRC10 and UKMRC11 could have suppressed neuropeptide-Y secretion, resulting in a relatively unchanged secretory pattern throughout the 2 h postprandial period. This concurs with a cell line study, which observed that insulin inhibits neuropeptide-Y neuronal activity in the hypothalamic arcuate nucleus, consequently reducing the neuropeptide-Y secretions [49]. In contrast, another cell line study elucidated the inhibitory effects of this hormone on insulin secretions via the G-protein coupled receptor pathway [50], suggesting that bi-directional relationship exists between these two hormones. The higher neuropeptide-Y responses observed after UKMRC9 and Basmati consumption could perhaps explain the lower postprandial insulin trends. However, whether or not these elevations would trigger physiological feeling of hunger in humans remains to be explored.

## 5. Conclusions

Amongst the three crossbred red rice variants and the comparator Thai red rice, only UKMRC9 facilitated the most desirable glucometabolic responses, particularly the acute postprandial insulin sensitivity. Since rice is a significant cereal option for most Asians, replacing white rice with a red rice displaying the characteristics of UKMRC9 becomes a critical factor in lowering dietary glycaemic load and insulin surge patterns attributed to the aetiology of metabolic syndrome. The incorporation of culturally acceptable, high-quality staple foods in substitution for refined grains is in line with recent international dietary guidelines. A robust intervention trial would serve to answer if there is/are any health-bearing benefits of replacing white rice with a low-GI, polyphenol-rich red rice on glucometabolic markers among Malaysians, particularly those with diabetes or at high risk for diabetes.

## Figures and Tables

**Table 1:** Proximate nutrient composition (% by dry weight basis) ^†^ and cooking characteristics of test rice.

Test Rice	Energy (kcal)	Total CHO (%)	Crude Protein (%)	Crude Lipid (%)	TDF (%)	Total Ash (%)	Available CHO (%)	Amylose (%)	TPC (% mg GAE)	Weight of Raw Rice (g) ^‡^	Weight of Cooked Rice (g) ^‡^		Cooking Time (min) ^§^
Crossbred red rice													
UKMRC9	364 ± 1 ^a^	78.4 ± 0.10 ^b^	8.23 ± 0.12 ^a^	1.93 ± 0.26 ^a^	4.96 ± 0.16 ^a^	1.32 ± 0.01 ^a^	73.4 ± 0.26 ^c^	19.8 ± 0.35 ^a,b^	61.4 ± 2.59 ^b^	68.1	178.9	44	
UKMRC10	355 ± 0 ^b,c^	76.2 ± 0.05 ^c^	7.44 ± 0.05 ^a,b^	2.20 ± 0.02 ^a^	4.25 ± 0.19 ^b^	1.30 ± 0.02 ^a^	71.9 ± 0.14 ^d^	19.0 ± 1.41 ^a,b^	81.7 ± 1.25 ^a^	69.5	181.1	45	
UKMRC11	354 ± 0 ^c^	76.7 ± 0.57 ^c^	7.03 ± 0.55 ^b^	2.17 ± 0.01 ^a^	3.84 ± 0.14 ^b,c^	1.30 ± 0.03 ^a^	72.8 ± 0.42 ^c,d^	17.5 ± 0.71 ^b^	55.2 ± 2.03 ^b^	68.7	170.2	41	
Commercial rice													
Thai red	356 ± 1 ^b^	76.5 ± 0.20 ^c^	7.76 ± 0.15 ^a,b^	2.14 ± 0.08 ^a^	3.70 ± 0.09 ^c^	1.15 ± 0.00 ^b^	72.8 ± 0.30 ^c,d^	18.0 ± 1.41 ^b^	81.9 ± 3.53 ^a^	68.7	174.2	40	
Basmati	354 ± 1 ^c^	79.2 ± 0.26 ^a,b^	8.25 ± 0.36 ^a^	0.47 ± 0.10 ^b^	1.96 ± 0.08 ^d^	0.42 ± 0.01 ^c^	77.3 ± 0.34 ^b^	21.5 ± 0.71 ^a,b^	29.8 ± 1.60 ^c^	64.7	188.3	26	
Jasmine	349 ± 0 ^d^	79.6 ± 0.30 ^a^	6.98 ± 0.16 ^b^	0.26 ± 0.07 ^b^	0.24 ± 0.01 ^e^	0.15 ± 0.00 ^d^	79.4 ± 0.31 ^a^	23.0 ± 1.41 ^a^	16.2 ± 1.51 ^c^	62.9	180.3	32	

^†^ Values are expressed as mean ± standard deviation with each variety analyzed in duplicate samples (*n* = 2). Values in the same column not superscripted by the same letter are significantly different, *p* < 0.05 (univariate analyses of variance, followed by Tukey’s *post hoc* test); **^‡^** Raw and cooked rice weights were based on 50 g available CHO content; ^§^ Cooking time was recorded from the time the electric rice cooker was switched on to the time it automatically turned off. CHO, carbohydrate; TDF, total dietary fiber; TPC, total phenolic content.

**Table 2:** Kinetic markers of postprandial glycaemic and insulin responses.

Test Diet	GLU-C_max_ (mmol/L) ^1^	GLU-∆_peak_ (mmol/L) ^1^	GLU-T_max_ (min) ^1^	GLU-T_∆0_ (min) ^1^	GI (%) ^1^	GI Category ^2^	INS-C_max_ (mU/L) ^1^	INS-∆_peak_ (mU/L) ^1^	IGI/HOMA-IR (×10^2^) ^1^	IGI/FPI ^1^	Matsuda Index ^1^	II (%) ^1^
GLU std.	8.45 ± 0.34	3.43 ± 0.28	35.0 ± 3.4	121.4 ± 10.2	100	-	96.2 ± 9.96	90.3 ± 9.80	1.60 ± 0.03	4.91 ± 0.87	6.17 ± 0.64	100
Crossbred red rice												
UKMRC9	7.34 ± 0.27	2.36 ± 0.23	37.5 ± 2.3 ^a,b^	85.8 ± 10.2	46 ± 7.7	Low	56.4 ± 5.61	51.5 ± 5.57	1.37 ± 0.03	4.11 ± 0.74	9.97 ± 0.78	51 ± 5.3 ^a^
UKMRC10	8.01 ± 0.38	2.98 ± 0.34	40.0 ± 3.4 ^a,b^	110.2 ± 13.9	59 ± 8.8	Intermediate	63.3 ± 6.69	57.7 ± 6.37	1.41 ± 0.03	4.35 ± 0.98	8.50 ± 0.83	69 ± 7.7 ^a,b^
UKMRC11	8.10 ± 0.24	3.08 ± 0.20	38.8 ± 2.2 ^a,b^	120.5 ± 13.8	63 ± 8.6	Intermediate	84.9 ± 10.1	77.1 ± 9.99	1.59 ± 0.04	4.84 ± 1.19	7.27 ± 0.67	69 ± 5.9 ^a,b^*
Commercial rice												
Thai red	7.53 ± 0.20	2.60 ± 0.20	38.8 ± 3.4 ^a,b^	100.5 ± 13.0	55 ± 8.6	Intermediate	67.7 ± 6.21	61.5 ± 5.97	1.43 ± 0.03	4.33 ± 0.85	8.37 ± 0.78	59 ± 4.0 ^a,b^
Basmati	7.37 ± 0.16	2.41 ± 0.12	35.0 ± 2.1 ^a^	99.6 ± 10.4	50 ± 5.8	Low	56.7 ± 4.19	50.9 ± 3.97	1.17 ± 0.02	3.59 ± 0.52	9.08 ± 0.75	52 ± 5.3 ^a,b^
Jasmine	8.15 ± 0.24	3.13 ± 0.25	47.5 ± 2.5 ^b^	136.5 ± 11.6	77 ± 7.3	High	78.7 ± 11.6	72.9 ± 11.6	1.33 ± 0.02	4.08 ± 0.74	7.04 ± 0.53	76 ± 7.1 ^b^
*p*-value (η_p_^2^) ^§^	0.074 ^ns^ (0.138)	0.063 ^ns^ (0.144)	0.043 (0.156)	0.075 ^ns^ (0.138)	0.093 ^ns^ (0.132)	-	0.061 ^ns^ (0.145)	0.069 ^ns^ (0.141)	0.952 ^ns^ (0.017)	0.947 ^ns^ (0.018)	0.058 ^ns^ (0.147)	0.018 (0.186)

^1^ Values are expressed as mean ± SEM. Mean values within the same column superscripted by different letters were significantly different (^§^ *p* < 0.05, univariate analysis of variance with Tukey’s *post hoc* test; ns = not significant between rice types); ^2^ GI values were categorized as low (<55), intermediate (55–70) and high (>70) [19]; * One subject was excluded (*n* = 11) as individual GI and II values >2 standard deviations from the respective mean GI and II scores [25]; GI, glycaemic index; GLU-C_max_, maximum concentration of postprandial plasma glucose; GLU-∆_peak_, incremental glucose peak; GLU-T_max_, time taken to reach GLU-C_max_; GLU-T_∆0_, time taken for returning of plasma glucose to baseline levels; IGI/FPI, ratio of insulinogenic index to fasting plasma insulin; IGI/HOMA-IR, ratio of insulinogenic index to homeostatic model assessment of insulin resistance; II, insulin index; INS-C_max_, maximum concentration of postprandial plasma insulin; INS-∆_peak_, incremental insulin peak; η_p_^2^ = partial eta-squared, 0.01, 0.06 and 0.14 were used to denote small, moderate and large differences in measured outcomes, respectively.

**Table 3:** Correlation between nutrient content, cooking characteristics, glycaemic and insulin indices of test rice.

	Glycaemic Index		Insulin Index	
	Pearson’s *r*	*p*-Value	Pearson’s *r*	*p*-Value
*Rice nutrients*				
Crude protein	−0.357	0.002 **	−0.385	0.001 **
Crude lipid	−0.133	0.268	0.006	0.958
Total dietary fiber	−0.237	0.047 *	−0.134	0.263
Total ash	−0.172	0.152	−0.037	0.756
Total amylose	0.093	0.441	−0.061	0.613
Total phenolic content	−0.158	0.189	−0.057	0.637
*Cooking characteristics*				
Cooking time	−0.060	0.622	0.035	0.772
Rice-to-water ratio	−0.175	0.145	−0.093	0.442
Meal serving size	−0.082	0.499	−0.145	0.227

* *p* < 0.05, ** *p* < 0.01 as per Pearson’s bivariate correlation test. The strength of correlation is defined as: trivial (0–0.1), weak (0.1–0.3), moderate (0.4–0.6), strong (0.7–0.9) and perfect (1.0).

## Supplementary Materials

The following are available online at http://www.mdpi.com/2072-6643/8/5/308/s1, Table S1: Postprandial plasma motilin, neuropeptide-Y and orexin-A responses (ng/mL) at baseline and 2 h post-consumption of test rice and glucose standard (mean ± SEM).

## Abbreviations

The following abbreviations are used in this manuscript:

CV	coefficient of variation
EIA	enzyme immunoassay
FPI	fasting plasma insulin
GI	glycaemic index
GLM	general linear model
HOMA-IR	homeostatic model assessment of insulin resistance
IAUC	incremental area-under-the-curve
IGI	insulinogenic index
II	insulin index
SEM	standard error of the mean
