# Supplementary material for: Evaluating Crossbred Red Rice Variants for Postprandial Glucometabolic Responses: A Comparison with Commercial Varieties
Source: Nutrients. 2016 May 20;8(5):308. doi: 10.3390/nu8050308 (PMC4882720; doi:10.3390/nu8050308)
Supplement: Supplementary file 1 [file nutrients-08-00308-s001.docx]

Supplementary Materials: Evaluating Crossbred Red Rice Variants for Postprandial Glucometabolic Responses: A Comparison with Commercial Varieties

Chee-Hee Se, Khun-Aik Chuah, Ankitta Mishra, Ratnam Wickneswari and Tilakavati Karupaiah

**Table S1.** Postprandial plasma motilin, neuropeptide-Y and orexin-A responses (ng/mL) at baseline and 2 h post-consumption of test rice and glucose standard (mean ± SEM).

| **Diet** | **Time (Minutes Postprandial)** | | | | | ***p*-Value *** |
| --- | --- | --- | --- | --- | --- | --- |
|  | **0** | **30** | **60** | **90** | **120** |  |
| *Motilin* | | | | | |  |
| GLU std. | 1.26 ± 0.07 ^a^ | 1.49 ± 0.08 ^a^ | 1.59 ± 0.15 ^a^ | 1.41 ± 0.13 ^a^ | 1.31 ± 0.09 ^a^ | 0.006 |
| UKMRC9 | 1.26 ± 0.07 ^a^ | 1.47 ± 0.14 ^a^ | 1.23 ± 0.12 ^a^ | 1.45 ± 0.10 ^a^ | 1.24 ± 0.09 ^a^ | 0.148 |
| UKMRC10 | 1.34 ± 0.08 ^a,b^ | 1.43 ± 0.13 ^a,b^ | 1.31 ± 0.11 ^a^ | 1.79 ± 0.17 ^b^ | 1.13 ± 0.08 ^a^ | 0.001 |
| UKMRC11 | 1.26 ± 0.12 ^a^ | 1.43 ± 0.13 ^a,b^ | 1.15 ± 0.09 ^a^ | 1.60 ± 0.11^b^ | 1.07 ± 0.07 ^a^ | 0.001 |
| Thai red | 1.44 ± 0.08 ^a,c^ | 1.83 ± 0.09 ^b^ | 1.37 ± 0.13 ^a^ | 1.54 ± 0.15 ^b,c^ | 1.28 ± 0.05 ^a,c^ | 0.030 |
| Basmati | 1.39 ± 0.09 ^a^ | 1.59 ± 0.12 ^a^ | 1.50 ± 0.12 ^a^ | 1.56 ± 0.11 ^a^ | 1.40 ± 0.06 ^a^ | 0.441 |
| Jasmine | 1.14 ± 0.09 ^a^ | 1.33 ± 0.08 ^a^ | 1.28 ± 0.07 ^a^ | 1.41 ± 0.14 ^a^ | 1.13 ± 0.06 ^a^ | 0.216 |
| *Neuropeptide-Y* | | | | | |  |
| GLU std. | 1.13 ± 0.06 ^a,b^ | 0.92 ± 0.05 ^a^ | 1.01 ± 0.06 ^a,b^ | 1.06 ± 0.06 ^a^ | 1.31 ± 0.09 ^b^ | <0.001 |
| UKMRC9 | 1.06 ± 0.04 ^a^ | 1.27 ± 0.06 ^b^ | 1.28 ± 0.15 ^a,b^ | 1.59 ± 0.17 ^a,b^ | 1.08 ± 0.05 ^a,b^ | 0.038 |
| UKMRC10 | 1.13 ± 0.08 | 1.13 ± 0.07 | 1.22 ± 0.08 | 1.25 ± 0.05 | 1.15 ± 0.09 | 0.449 |
| UKMRC11 | 1.10 ± 0.05 | 1.04 ± 0.03 | 1.05 ± 0.03 | 1.16 ± 0.05 | 1.14 ± 0.08 | 0.308 |
| Thai red | 1.02 ± 0.06 ^a^ | 0.88 ± 0.07 ^b^ | 1.03 ± 0.05 ^a^ | 1.13 ± 0.06 ^a^ | 1.01 ± 0.05 ^a^ | 0.027 |
| Basmati | 1.02 ± 0.06 | 1.12 ± 0.07 | 1.22 ± 0.15 | 1.60 ± 0.19 | 1.25 ± 0.15 | 0.066 |
| Jasmine | 1.18 ± 0.07 | 1.37 ± 0.06 | 1.19 ± 0.07 | 1.54 ± 0.13 | 1.27 ± 0.16 | 0.058 |
| *Orexin-A* | | | | | | |
| GLU std. | 1.24 ± 0.14 ^a^ | 1.28 ± 0.12 ^a,b^ | 1.53 ± 0.08 ^a,b^ | 1.70 ± 0.24 ^a,b^ | 1.77 ± 0.19 ^b^ | 0.004 |
| UKMRC9 | 1.04 ± 0.06 ^a^ | 1.47 ± 0.16 ^b^ | 1.10 ± 0.14 ^a^ | 1.50 ± 0.18 ^b^ | 0.97 ± 0.06 ^a^ | <0.001 |
| UKMRC10 | 0.88 ± 0.07 ^a^ | 1.34 ± 0.14 ^b,c^ | 1.03 ± 0.06 ^a,b^ | 1.36 ± 0.10 ^c^ | 0.83 ± 0.07 ^a^ | <0.001 |
| UKMRC11 | 1.19 ± 0.11 ^a,c^ | 1.66 ± 0.11 ^b^ | 1.23 ± 0.17 ^a,b,c^ | 1.42 ± 0.09 ^a,b^ | 1.14 ± 0.07 ^c^ | 0.001 |
| Thai red | 1.33 ± 0.14 ^a,c^ | 1.98 ± 0.16 ^b,c^ | 1.34 ± 0.10 ^a^ | 1.76 ± 0.13 ^c^ | 1.26 ± 0.12 ^a,b,c^ | <0.001 |
| Basmati | 1.11 ± 0.06 ^a,d^ | 1.47 ± 0.12 ^b,d^ | 1.03 ± 0.07 ^a,d^ | 1.53 ± 0.10 ^b,c^ | 1.08 ± 0.05 ^d^ | <0.001 |
| Jasmine | 0.99 ± 0.07 ^a^ | 1.59 ± 0.13 ^b^ | 1.09 ± 0.11 ^a,b^ | 1.61 ± 0.16 ^a,b^ | 1.14 ± 0.09 ^a,b^ | <0.001 |

Data were log-transformed before subjected to one-way repeated-measures ANOVA, followed by Bonferroni *post hoc* comparisons when the tested time effect was significant (* *p* < 0.05). Values within the same row superscripted by the same alphabets were not significantly different (*p* > 0.05).
